# From Being Moved to Being Still: Kama Muta Reduces Postural Sway Velocity

**DOI:** 10.1002/brb3.70897

**Published:** 2025-09-23

**Authors:** Maria Meisel, Philipp Hofmann, Petra Jansen

**Affiliations:** ^1^ Faculty of Human Sciences University of Regensburg Regensburg Germany

**Keywords:** emotional embodiment, kama muta, motor control, postural sway

## Abstract

**Objectives:**

Grounded in embodiment theory, this exploratory study examines whether kama muta (as being moved/touched) elicits measurable changes in postural sway (subtle body movement even during stillness) reflecting an emotional–motor connection.

**Methods:**

Data were collected from 87 university students (aged 18–43 years, *M* = 22.22, *SD* = 3.20). Participants viewed six video clips (kama muta and neutral condition) while standing on a force plate that recorded their postural sway. After each video, participants rated their feelings of being moved on a 5‐point Likert scale.

**Results:**

Kama muta was associated with a significant reduction in sway velocity compared to the neutral condition (*p* = 0.003), with no significant differences in mean amplitude (*p* = 0.31). Sway velocity and amplitude decreased significantly in the second half of the kama muta condition (*p* < 0.001), indicating intensification of the emotional effect. No significant correlations emerged between self‐reported emotional ratings and sway metrics (*p* > 0.05).

**Conclusion:**

Findings indicate a possible stabilizing effect of kama muta on postural control, promoting physical grounding during emotional experiences. While the homogeneous sample limits generalizability, these results enhance our understanding of emotional embodiment and suggest potential applications for fostering emotional and social connection. Future research should explore these mechanisms by including comprehensive emotion assessments and additional measures to identify muscular activation patterns.

## Graphical abstract

Kama muta describes the emotion of being moved or touched. This study demonstrated that kama muta can stabilize posture because a reduction in sway velocity can be observed.

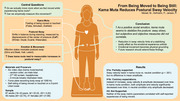

## 1 Introduction

Emotions and physical movement are deeply interconnected—not only at the behavioral level but also in neural and physiological terms. Barrett (2018) emphasizes that emotions are constructed experiences shaped by bodily states, while Braine and Georges (2023) describe how emotions directly engage motor circuits. Similarly, De Gelder et al. (2015) highlight how emotions are perceived and interpreted through body expressions, and Schönfeld and Wojtecki (2019) link emotional oscillations in the amygdala to changes in movement readiness. Recent theories highlight emotions embedded within relational contexts, influencing cognitive interpretation and bodily responses (Barrett 2018; Fiske 2020). This shift opens new avenues for exploring how specific emotions manifest through physical expression. Kama muta—derived from Sanskrit and describing the experience of “being moved” or “being touched”—is deeply associated with communal sharing and moments of social bonding (Fiske 2020). The term refers to a specific emotional experience rooted in a sense of unity and togetherness, often accompanied by bodily sensations such as warmth in the chest, tears, or a lump in the throat. This complex experience also suggests a subjective awareness of a motor change or perceived bodily response. In line with the theory of linguistic relativity (Sapir 1956; Whorf 1956)—which suggests that the words we use can shape our perception and interpretation of experiences—and with embodiment theory, which posits that emotional and bodily movements are interrelated, this study explores whether *being moved* reflects more than a metaphor—namely, an embodied phenomenon that can be captured empirically.

### 1.1 Kama Muta as a Specific “Moved” Emotion

The concept of kama muta captures how emotions can arise from intensified feelings of belonging and unity (Fiske 2020). This emotion manifests during intense social connection, love, or unity and is considered a unique self‐transcendent emotion distinct from other positive emotions. Unlike awe, which may evoke chills or goosebumps, or elevation, which leads to sensations of moral uplift, kama muta is typically accompanied by physical sensations such as warmth in the chest, tears, and sometimes a lump in the throat (Zickfeld et al. 2020). The experience is intense, with a rapid onset and brief duration, peaking quickly and subsiding shortly thereafter (Schubert et al. 2018). Kama muta is a universal phenomenon (Zickfeld et al. 2019).

The physiological markers of the kama muta experience have been thoroughly investigated. In one study (Zickfeld et al. 2020), physiological responses were measured in participants while they watched emotionally moving videos. Kama muta emotion was found to correlate with heightened skin conductance, chest area warmth, piloerection, increased zygomaticus activity (indicative of smiling), and changes in heart rate. These findings support the view that kama muta affects not only emotional awareness but also bodily systems.

From the embodiment perspective, Meier et al. (2012) suggest that emotions could subtly influence motor control, from posture changes to coordination shifts. This suggests that when people feel emotionally moved, corresponding observable movements may occur, reflecting or even enhancing the emotional experience. Although substantial advances have been made in understanding the physiological markers of kama muta, the role of motor control remains largely unexplored. The current study addresses this gap by investigating how the body responds through movement when we experience “being moved” by utilizing posturography and center of pressure (CoP) analyses. This approach will allow us to objectively assess whether the subjective feeling of being moved corresponds to measurable physical changes.

### 1.2 Postural Sway as a “Moved” Body Sensation

Body's postural sway is defined as the natural, continuous shifting of the center of gravity even when a person is standing still. This sway is often quantified using a force plate, which detects shifts in the body's CoP by measuring the vertical ground reaction forces (Doyle et al. 2007; Duarte and Watanabe 2023; Fredrickson and Levenson 1998; Prieto et al. 1996; Stins and Beek 2007). Key metrics in posturography include sway amplitude and sway velocity. Winter (1995) and Doyle et al. (2007) have shown how these measures can detect minimal variations in balance under emotional or cognitive load. Sway amplitude is typically defined as the distance between the maximum and minimum CoP displacement in each direction (anterior–posterior and medial–lateral), providing a measure of the spatial range of sway (Duarte and Freitas 2010). Sway velocity, by contrast, measures the speed at which these movements occur, reflecting the dynamic control mechanisms of balance and postural adjustments. These metrics capture subtle, often unconscious balance corrections and provide insight into how the body responds to emotional stimuli (Duarte and Watanabe 2023). By analyzing sway amplitude and velocity, we could explore how kama muta might subtly influence postural stability and balance, hinting at an emotional–motor connection (Duarte and Watanabe 2023; Winter 1995).

In psychological and behavioral studies, force plates help quantify movement patterns in response to emotional stimuli, revealing how emotions influence physical movement. For example, unpleasant images have been shown to induce postural freezing, characterized by a reduction in sway amplitude and velocity—a defensive response to perceived threats (Roelofs et al. 2010). Hillman et al. (2004) found that affective picture viewing alters posture, suggesting a motivational component in postural responses. Similarly, Facchinetti et al. (2006) and Roelofs et al. (2010) observed postural freezing in response to threatening stimuli—a defensive mechanism modulated by emotional salience.

### 1.3 Relation Between Emotional and Bodily Movements

The relationship between emotional states and bodily movement is complex, with evidence suggesting that emotional arousal modulates postural sway by engaging the autonomic nervous system to influence motor functions (Fredrickson and Levenson 1998; Hall et al. 2023; Hillman et al. 2004; Hofmann et al. 2021). Studies show that emotional arousal can prepare the body for specific responses, such as approach or avoidance, by affecting balance and postural adjustments. This interplay between affective and motor systems provides a promising framework for understanding embodied emotions. Research by Facchinetti et al. (2006) highlights that threatening images, for example, elicited a freezing response with reduced sway amplitude, especially along the medial–lateral axis. In contrast, prosocial stimuli like images of smiling babies influenced posture differently, causing reduced sway primarily along the anterior–posterior axis.

Further research has also examined how positive emotions impact postural control. Lelard et al. (2014) found that exposure to pleasant stimuli increased sway amplitude along the anterior–posterior axis, reflecting an approach tendency consistent with activating the appetitive motivational system. Similarly, Gea et al. (2014) showed that happy and pain‐related facial expressions led to increased sway in the anterior–posterior direction compared to neutral expressions, highlighting how empathetic reactions can influence bodily movements.

Maki and McIlroy (1996) investigated the effects of arousal and attention on postural control, finding that tasks inducing higher arousal levels, such as mental arithmetic, led to increased forward‐leaning and changes in muscle activation patterns. This suggests that physiological arousal can influence postural adjustments, potentially preparing the body for action in response to emotional stimuli. Although kama muta is distinct from high‐arousal emotions like fear or excitement, it also involves a degree of physiological arousal, often manifested as a warm sensation in the chest area, changes in heart rate, and physical movements like tears or smiles (Zickfeld et al. 2020).

Emotional stimuli with affective content can also shape motor behavior. Hillman et al. (2004) observed that viewing unpleasant images led to sex‐specific postural adjustments: females exhibited increased backward sway (withdrawal behavior), while males showed forward sway (approach tendencies). Gea et al. (2014) found that viewing faces expressing pain or happiness elicited similar postural sway changes, suggesting that highly salient emotional cues can modulate motor responses. This emphasizes the social relevance of emotional expressions and their capacity to affect an observer's physiological state and motor behavior. These findings align with the biphasic theory of emotion, which posits that emotions are organized around appetitive (approach) and defensive (withdrawal) motivational systems (Lang 1995). Further expanding on this, Stins and Beek (2007) reported that unpleasant images, especially those depicting mutilation, resulted in reduced postural sway path length during unipedal stance—a freezing response consistent with heightened defensive motivation (Roelofs et al. 2010). Horslen and Carpenter (2011) researched the effects of arousal and valence on postural control, demonstrating that arousal independently increased the frequency of CoP displacements, regardless of the emotional content's valence.

Despite these findings on how various emotional states modulate postural sway, there is no direct evidence on whether a strongly social and positively valenced emotion such as kama muta might lead to measurable bodily changes. This study addresses this gap by linking the subjective experience of being moved to objective measures of bodily sway.

### 1.4 Present Study

While past research has established kama muta as a universal emotion that fosters communal bonds and evokes specific physiological responses, this study extends these findings by examining how kama muta corresponds with physical movement. Specifically, we investigate whether experiences of kama muta, elicited by carefully selected emotional video stimuli, lead to measurable changes in postural sway as recorded by CoP displacements.

In particular, we take an exploratory stance, as kama muta theory itself does not specify if or how body sway should shift during a moving experience. Nonetheless, drawing on findings that other emotions, especially intense ones, can lead to reduced sway velocity (reactions like freezing) or altered sway amplitude, we hypothesize that kama muta—a self‐transcendent, socially bonding emotion—might also induce measurable modifications in sway.

**H1a**. The emotional experience of being moved will change sway metrics, such as sway velocity and mean amplitude, when compared to neutral stimuli.
**H1b**. The effect of kama muta on postural sway will be more pronounced in the latter half of the recording, indicating that the emotional impact evolves and may correspond with the intensification of the emotional experience.
**H2**. There will be a positive correlation between participants’ self‐reported experiences of being moved and CoP data, particularly sway velocity and amplitude, highlighting the relationship between subjective emotional experiences and physical responses.

## 2 Materials and Methods

This study used a repeated measurement design without blinding to test three hypotheses. To ensure controlled conditions, the research was conducted individually for each participant in the biomechanical laboratory at the Institute for Sport Sciences, University of Regensburg, Germany. The same experimenter conducted all sessions. The study was performed in accordance with the principles of the Declaration of Helsinki and was approved by the ethical committee of the University of Regensburg (Approval Number: 22–2892_1‐101). Participants received information about the study via a recruitment email sent through the department's internal mailing list and on site before the testing session. Privacy policies concerning data handling were provided in a written consent form.

### 2.1 Sample Size Calculations

For each hypothesis, a separate a priori G*Power analysis was performed (Faul et al. 2007). For all three hypotheses, the alpha level is set at *p* = 0.05 and the desired power at 1 − beta = 0.8; for H1a, with the alpha level set at *p* = 0.05 and power at 1 − beta = 0.8, a small to medium effect size of 0.35 is being expected, resulting in a sample size of *N* = 67. The effect size is derived from Hillman et al. (2004) and Horslen and Carpenter (2011). Due to the lack of suitable research connecting kama muta with body sway, a connection between arousal levels and experienced kama muta feeling has been used to estimate a medium‐sized effect for H1b, based on a study linking kama muta and arousal, done by Zickfeld et al. (2020). The alpha error remains *p* = 0.05 with power at 1 − beta = 0.8. Based on these data, the estimated sample size is *N* = 34. For H2, employing a correlation method, a correlation coefficient of *R* ^2^ = 0.07 from Va'ez‐Mousavi and Osanlu (2011) was utilized in the G*Power calculation. Maintaining the alpha level at *p* = 0.05 and power at 1 − beta = 0.8, the required sample size for this hypothesis is *N* = 87. So, the total dataset aimed for this study is *N* = 87.

### 2.2 Participants

Participants were healthy students recruited in the summer 2024 at the University of Regensburg. All participants received course credit for their participation. Exclusion criteria included diseases or injuries affecting balance and acute psychological issues; these criteria were screened before the recruitment. A total of 92 participants were tested. The first three participants were excluded, as their data were used to finalize the experimental setup in the laboratory. Additionally, force plate data from two participants were lost due to technical issues. The final sample consisted of 87 participants in total (age range: 18–43, *M*_age_ = 22.22, *SD*_age_ = 3.20). The sample included 46 male participants (age range: 19–31, *M*_age_ = 22.70, *SD*_age_ = 2.36) and 41 female participants (age range: 18–43, *M*_age_ = 21.68, *SD*_age_ = 3.90).

### 2.3 Materials

The experiment was programmed using PsychoPy software (version 2023.2.3) (Peirce et al. 2019). Video stimuli were presented on a 27‐inch Philips 272S1M LCD monitor with a Full HD resolution of 1920×1080 pixels, mounted on an adjustable platform to match the participant's height prior to the experimental start. Audio was delivered via over‐ear headphones (Bose, QuietComfort) selected to minimize external distractions and ensure clear audio delivery of the video stimuli. Participants watched the video clips while standing in a bipedal position on an AMTI OR6‐7‐2000 force plate, which recorded CoP displacements at a sampling rate of 1000 Hz. Two parameters of CoP, namely sway amplitude and sway velocity, were computed based on these recordings. Vicon Nexus software for motion capture (version 2.13.0) (Vicon Motion Systems n.d.) was used as a user interface to manage force plate recordings.

Six video clips were presented individually in random order (duration is indicated in parentheses): “Thai Medicine” (2:56), “Elephant Rescue” (2:56), “Christian the Lion” (2:29), “Two Orphans” (2:59), “Thai Altruism” (2:59), and “Searching for Bobby Fischer” (3:10). All materials were in English or subtitled in English. Before implementation, all videos were reviewed by the research team to confirm their suitability and clarity. We chose not to modify the clips by translating them into German to maintain the authenticity of their original form and ensure comparability with existing research. Participants were university students with solid English language skills, sufficient to understand the content of the clips easily. The first five videos have been previously validated and approved for eliciting kama muta (Schubert et al. 2018). These video clips are well‐known and frequently used in various kama muta studies. “Searching for Bobby Fischer” was used as a neutral stimulus based on prior validation work by İyilikci et al. (2023). In their comprehensive assessment of different film clips across multiple emotion categories, the video “Searching for Bobby Fisher” consistently elicited low mean ratings for arousal and low intensities of discrete emotions, identifying this video clip as the least emotionally charged among the 104. Although no stimuli can be perfectly neutral for all viewers, this clip's minimal engagement relative to more clearly affective materials justifies its use as a baseline in our study.

See the Supporting Information published on OSF for the exact videos used in the research https://doi.org/10.17605/OSF.IO/P8ZQF.

After each video clip, participants completed a single‐item questionnaire asking them to rate their feeling of being moved or touched: “Please indicate how much you feel moved or touched after watching this video” (Schubert et al. 2018). Participants responded using a keyboard, rating their feelings on a 5‐point Likert scale, ranging from 1 (*Not moved/touched at all*) to 5 (*Extremely moved/touched*). Demographic information (such as age and sex) was collected at the start of the experiment to provide a statistical description of the sample.

### 2.4 Method to Calculate CoP Parameters

Sway velocity refers to the average speed at which the body's CoP moves during postural sway (Doyle et al. 2007). It is calculated as the total distance traveled by the CoP over time. Sway velocity reflects how fast postural adjustments occur and indicates the dynamic control mechanisms that help maintain balance. A higher sway velocity may indicate less stable postural control, as the body requires frequent and rapid adjustments to maintain balance. Conversely, a lower sway velocity suggests smoother and more controlled adjustments in posture. As given in Doyle et al. (2007), velocity is computed according to the formula:

$$\begin{array}{ccc} & & \mathrm{Sway}\;\mathrm{velocity}\\ & & \;=\frac{\sum_{n=1}^{N-1}{\left[{\left(x_{\mathrm{AP}\left(n+1\right)}-x_{\mathrm{AP}\left(n\right)}\right)}^{2}+{\left(x_{\mathrm{ML}\left(n+1\right)}-x_{\mathrm{ML}\left(n\right)}\right)}^{2}\right]}^{1/2}}{T}, \end{array}$$

where $x_{\mathrm{AP}(n)}$ and $x_{\mathrm{ML}(n)}$ represent CoP positions in the anterior–posterior (AP) and medial–lateral (ML) directions at time *n*. *N* is the total number of measurements. *T* is the total time of measurement.

Sway amplitude is typically computed as the root‐mean‐square deviation of the CoP positions from the mean CoP position (Quijoux et al. 2021). It measures how far the body sways on average, showing how much the CoP deviates from the mean position. Larger sway amplitudes suggest greater postural instability or body sway, while smaller amplitudes indicate a more stable position in space. In the present study, we calculated the mean amplitude as the average of the Euclidean distances from each CoP point to the mean CoP position. The formula we used to compute the mean amplitude is shown below:

$$\mathrm{Mean}\;\mathrm{amplitude}=\frac{1}{N}\sum_{i=1}^{N}\sqrt{{\left(C_{x}^{\left(i\right)}-\bar{C_{x}}\right)}^{2}+{\left(C_{y}^{\left(i\right)}-\bar{C_{y}}\right)}^{2}},$$

where $\mathrm{Co}P_{i}$ is the CoP position at time *i*, $\bar{\mathrm{CoP}}$ is the mean CoP position over the period of the measurement, and *N* is the total number of measurements or time intervals.

The CoP data were recorded continuously and segmented by trial. For each trial, sway velocity and mean amplitude were calculated as described and then averaged across all valid trials per condition. We chose this procedure to obtain stable per‐condition means for each participant.

### 2.5 Procedure

Upon arrival at the laboratory, participants provided written informed consent and were briefly familiarized with the procedure. They stood on the force plate with their feet positioned according to marked sections to ensure consistent standing conditions across all participants.

A vertical line was taped across the center of the force plate to divide it into equal halves, and a horizontal marker indicated the heel limit.

The monitor height was adjusted to eye level and approximately 60–62 cm from the participant's face. Headphones were provided at this point. Participants were instructed to remain still and to refrain from speaking during the video presentations. The room lights were turned off during the trial, making the monitor the primary light source. Each participant viewed six video clips, resulting in six trials in total. A trial is defined as presenting a single video stimulus, followed by a single‐item self‐report questionnaire (Schubert et al. 2018). To minimize fatigue and ensure consistent performance, 2‐minute breaks were instituted between each trial, as advised by Rottenberg et al. (2007). The self‐report questionnaire was administered during these breaks in a seated position. An experimental protocol was maintained throughout each session, documenting any noteworthy aspects of the experimental environment or participant behavior. This information was used for data exclusion purposes, such as when participants moved their arms, head, or shoulders, spoke aloud during the video, or exhibited other behaviors that could compromise the data. All trials with such compromising events were excluded from the final dataset. The total duration of the experiment was approximately 50 min.

### 2.6 Statistical Analysis

Before conducting statistical analyses, the data were preprocessed to ensure completeness. PsychoPy log data and force plate recordings were matched and compiled to create individual datasets for each participant. These were then combined into a comprehensive data frame, including all usable data points. Compromised trials, as documented in the lab protocol, were excluded. The mean values for postural sway metrics (mean amplitude and sway velocity) in each condition (kama muta and neutral/calm) were calculated. After this, statistical outliers were identified and removed, defined as values above the 75th percentile plus 1.5 times the interquartile range or below the 25th percentile minus 1.5 times the interquartile range. Following this step, the total means for both sway metrics in each condition were computed. We called these values *total mean*; they represent the average sway metrics per kama muta condition across all five trials. We implemented a two‐step outlier removal procedure to ensure robust estimation of sway metrics. First, we excluded trials identified as outliers at the individual trial level. A second outlier detection process was applied to the computed total mean values, and any statistical outliers at this stage were also excluded.

To evaluate H1a and H1b, paired‐sample *t*‐tests were conducted to compare dependent means. For H1a, postural sway metrics between the emotional condition (kama muta) and neutral video stimuli were compared. For H1b, the *t*‐tests assessed changes between the first and second halves of the recordings within the emotional (kama muta) and neutral/calm conditions. Assumptions of normality and homogeneity of variance were verified. Although we initially planned to use paired‐sample *t*‐tests, we also prespecified that nonparametric statistical methods (e.g., Wilcoxon signed‐rank test) would be applied if the normality assumption was violated. Consequently, we used Wilcoxon tests since the Shapiro–Wilk test indicated a violation of normality.

To explore H2, we initially planned to use Pearson's correlation coefficient to examine the relationship between self‐reported feelings of being moved and CoP metrics. However, Spearman's rank‐order correlation was used instead due to violations of the normality assumption (as tested with the Shapiro–Wilk test). This analysis aimed to quantify the association between subjective emotional experiences and objective physical responses.

### 2.7 Transparency and Openness

This study adhered to the Journal Article Reporting Standards (JARS) (Appelbaum et al. 2018). Sample size calculations were performed using G*Power (version 3.1.9.7) (Faul et al. 2007) and are detailed in Section 2.1. The experiment was preregistered at OSF https://doi.org/10.17605/OSF.IO/P8ZQF, and the design, hypotheses, and analysis plan were established prior to data collection.

All the supplementary materials are available at https://osf.io/p2wze/. This includes the G*Power calculation protocol (provided as a raw file for transparency), the final dataset with flagged outliers, a list of excluded trials with reasons, and references to the video stimuli materials. The PsychoPy software (version 2023.2.3) (Peirce et al. 2019) was used to program the experiment, while motion capture data were managed using Vicon Nexus software (version 2.13.0) (Vicon Motion Systems n.d.). Data curation was conducted using Python (version 3.9.6) (Python Software Foundation 2021). Both the PsychoPy experiment and the Python script for data curation are not included in the OSF repository but can be made available upon reasonable request. Statistical analyses were conducted using JASP (version 0.19.2) (JASP Team 2024), and data visualizations were created in R (version R 4.4.2) (R Core Team 2023).

## 3 Results

The analysis revealed no significant difference in postural sway amplitude between the emotional and neutral conditions (*M* = 5.127, *SD* = 1.125), *W* = 1299.00, *z* = –1.03, *p* = 0.31, 95% CI [–0.37, 0.12], with a Hodges–Lehmann estimate of –0.09 and a rank‐biserial correlation of *r*_B_ = –0.14 (*SE* = 0.13). These results indicate a small effect size (nonsignificant effect), suggesting consistent postural sway amplitude across both conditions.

**FIGURE 1:**
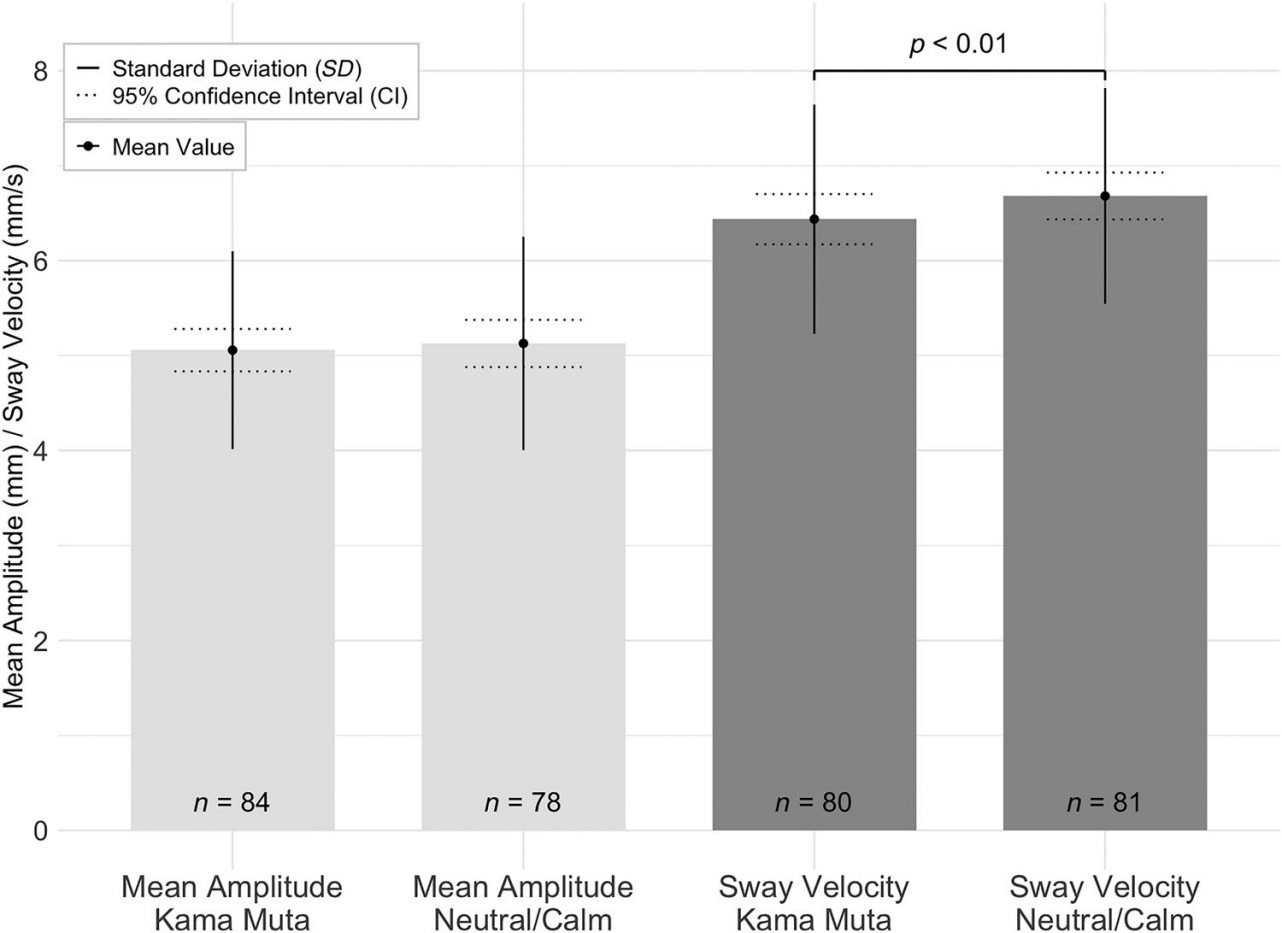
Mean amplitude (mm) and sway velocity (mm/s) across kama muta and neutral/calm conditions. The bracket above the paired bars for sway velocity indicates a significant difference (*p* < 0.01). Error bars represent the standard deviation (SD, solid lines) and 95% confidence interval (CI, dashed lines) for each condition. Sample sizes are displayed inside the bars (*N* = 84, 78, 80, and 81). See text for further statistical details.

However, there was a significant reduction in sway velocity in the kama muta condition (*M* = 6.44, *SD* = 1.21) compared to the neutral/calm condition (*M* = 6.68, *SD* = 1.14), *W* = 968.00, *z* = –2.99, *p* = 0.003, 95% CI [–0.58, –0.15], with a Hodges–Lehmann estimate of –0.054 and a rank‐biserial correlation of *r*_B_ = –0.387 (*SE* = 0.13), reflecting a medium‐to‐large effect size as shown in Figure 1. The results of the Wilcoxon signed‐rank test indicate that while postural sway amplitude remained consistent, sway velocity varied significantly in response to the kama muta stimuli compared to the neutral/calm condition.

The comparisons included the mean amplitude for the kama muta condition total mean (first half) paired with the mean amplitude for the kama muta condition total mean (second half), sway velocity for the kama muta condition total mean (first half) paired with sway velocity for the kama muta condition total mean (second half), mean amplitude for the neutral condition first half paired with mean amplitude for the neutral condition second half, and sway velocity for the neutral condition first half paired with sway velocity for the neutral condition second half.

**FIGURE 2:**
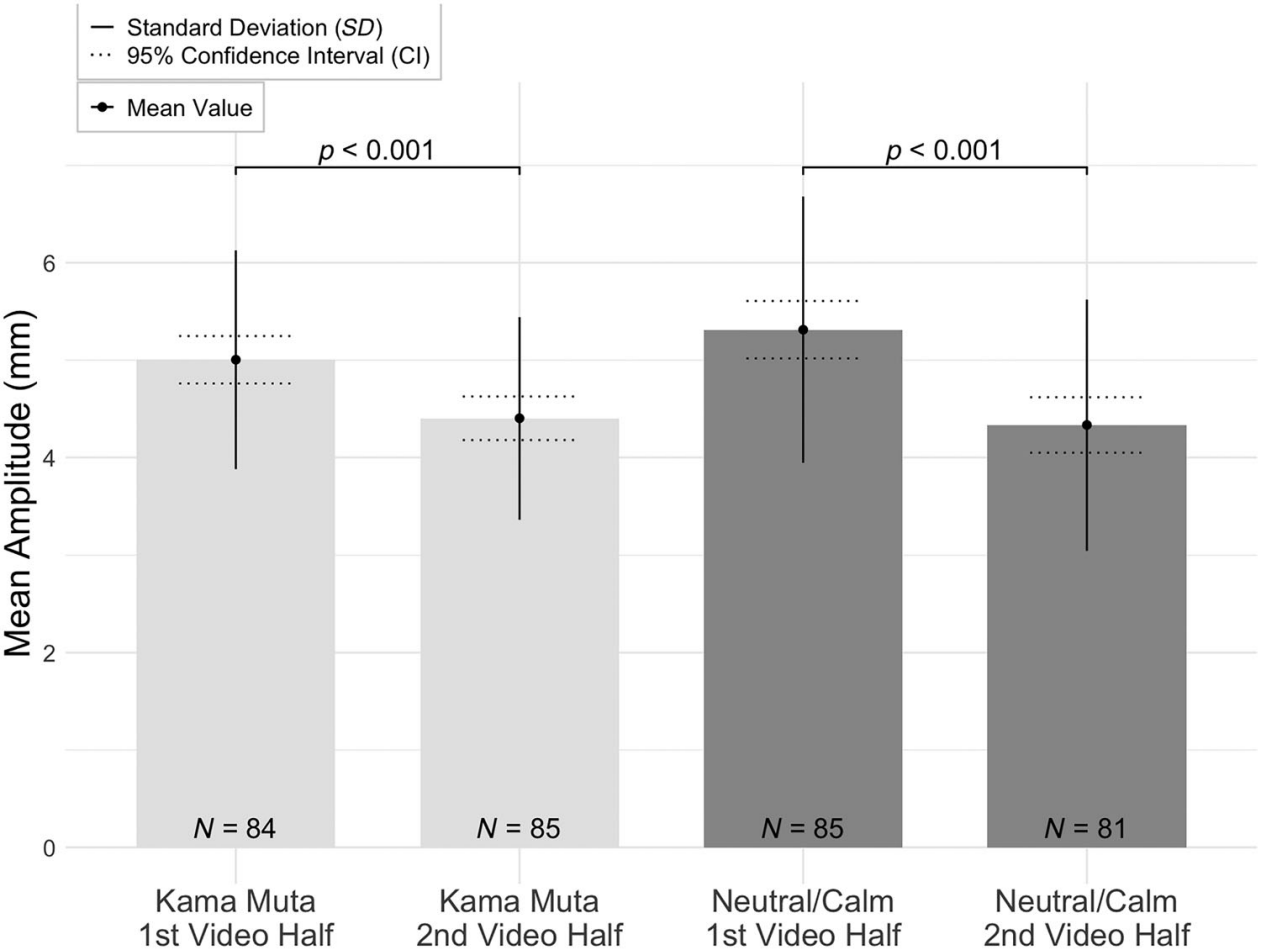
Mean amplitude (mm) across video's first and second halves in kama muta and neutral/calm conditions. Bars represent the mean amplitude for the first and second halves of the videos under kama muta and neutral/calm conditions. Error bars indicate standard deviation (SD) and 95% confidence interval (CI). Sample sizes are indicated inside each bar (*N* = 84, 85, 85, and 81). Results indicate significant differences between the first and second halves of the videos in both conditions (*p* < 0.001).

As shown in Figure 2, the mean amplitude was significantly lower in the second half of the kama muta condition (*M* = 4.40, *SD* = 1.04) compared to the first half (*M* = 5.00, *SD* = 1.12), *W* = 3455.00, *z* = 7.45, *p* < 0.001, 95% CI [0.90, 0.96], with a Hodges–Lehmann estimate of 0.60 and a large effect size indicated by a rank‐biserial correlation of *r*_B_ = 0.94 (*SE* = 0.13). In the neutral condition (Video 0), the mean amplitude between the first and second halves differed significantly as well (*M* = 4.33, *SD* = 1.29), *W* = 2900.00, *z* = 5.84, *p* < 0.001, 95% CI [0.61, 0.84], with a Hodges–Lehmann estimate of 0.85 and a large effect size (*r*_B_ = 0.75, *SE* = 0.13).

**FIGURE 3:**
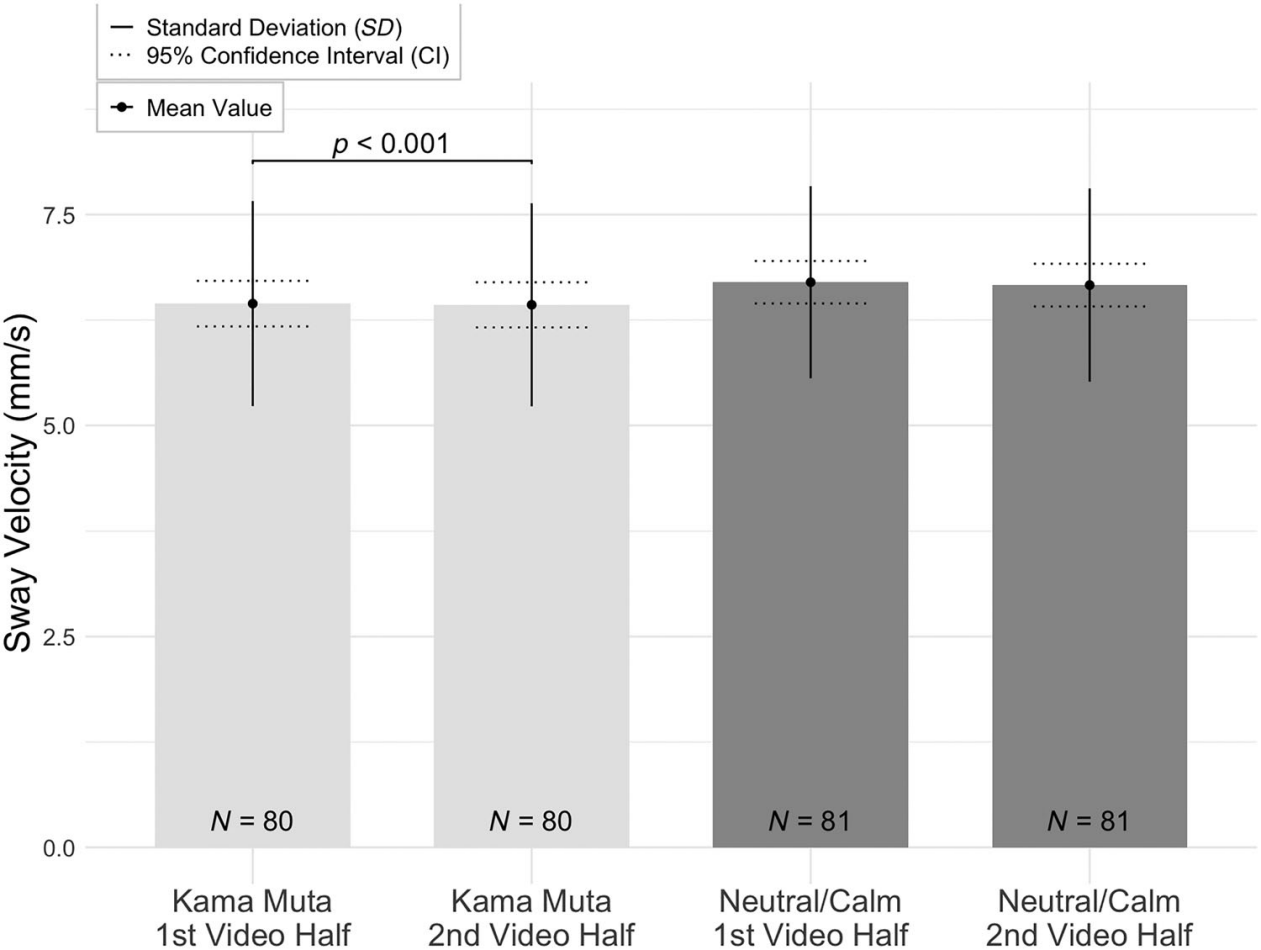
Sway velocity (mm/s) across video's first and second halves in kama muta and neutral/calm conditions. Bars represent the mean sway velocity for the first and second halves of the videos under kama muta and neutral/calm conditions. Error bars represent the standard deviation (SD) and 95% confidence interval (CI). Sample sizes are indicated inside each bar (*N* = 80, 80, 81, and 81). Results indicate significant difference between the first and second halves of the videos in kama muta condition (*p* < 0.001).

Sway velocity was also significantly lower in the second half (*M* = 6.43, *SD* = 1.20) compared to the first half (*M* = 6.44, *SD* = 1.22), *W* = 2388.00, *z* = 3.68, *p* < 0.001, 95% CI [0.26, 0.65], with a Hodges–Lehmann estimate of 0.01 and a medium effect size (*r*_B_ = 0.47, *SE* = 0.13). However, there was no significant difference in sway velocity between the first half (*M* = 6.70, *SD* = 1.14) and the second half (*M* = 6.66, *SD* = 1.15) of the neutral/calm condition, *W* = 2009.00, *z* = 1.64, *p* = 0.101, 95% CI [−0.04, 0.43], with a Hodges–Lehmann estimate of 0.001 and a small effect size (*r*_B_ = 0.21, *SE* = 0.13). The results are visualized in Figure 3.

Summing the results for H1b, we can state that mean amplitude and sway velocity decreased significantly in the second half of the kama muta condition, with larger effect sizes observed for mean amplitude. In the neutral/calm condition, only the mean amplitude significantly reduced in the second half, with no significant change in sway velocity.

For Hypotheses 2, Spearman's rho and corresponding *p*‐values for mean amplitude across each video condition are presented in Table 1, alongside additional information such as effect size or sample sizes. None of these correlations reached statistical significance (*p* > 0.05), indicating that self‐reported moving experiences were not consistently associated with mean amplitude across the emotional video conditions. For the neutral condition, there was also no significant correlation between mean amplitude and self‐reported experiences of kama muta. Effect sizes, expressed using Fisher's *z*, were small across all video conditions, further supporting the lack of a meaningful relationship between these variables. All values are given in Table 1.

**TABLE 1:** Spearman's correlations between mean amplitude and self‐reported experiences of being moved.

Correlated variables: Mean amplitude–Self‐reported kama muta	*N*	Spearman's rho	*p*	Fisher's *z*	*SE* effect size	95% CI [LL, UP]
Video 0 (neutral/calm)	76	−0.079	0.495	−0.080	0.117	[–0.30, 0.15]
Video 1 (kama muta)	78	0.110	0.336	0.111	0.116	[–0.12, 0.33]
Video 2 (kama muta)	80	−0.003	0.978	−0.003	0.113	[–0.22, 0.22]
Video 3 (kama muta)	80	−0.189	0.093	−0.191	0.115	[–0.39, 0.03]
Video 4 (kama muta)	80	−0.018	0.872	−0.018	0.113	[–0.24, 0.20]
Video 5 (kama muta)	78	−0.193	0.091	−0.195	0.116	[–0.40, 0.03]

*Note*: Table 1 reports the results of Spearman's rank‐order correlations between mean amplitude and self‐reported experiences of being moved for six video stimuli: one neutral (Video 0) and five kama muta videos (Videos 1–5). Spearman's rho, *p*‐values, and effect sizes are reported. *N* refers to the number of participants in each condition. Effect size is expressed using Fisher's *z*, with the corresponding standard error (*SE*).

Additional information, such as effect size or sample size, is provided in Table 2. Across the kama muta videos, correlations between sway velocity and self‐reported moving experiences were small and ranged from weakly negative to weakly positive. None of the correlations were statistically significant. No significant correlation was observed in the neutral/calm video condition. Effect sizes were small across all video conditions, indicating that self‐reported moving experiences were not consistently related to sway velocity (see Table 2).

**TABLE 2:** Spearman's correlations between sway velocity and self‐reported experiences of being moved.

Correlated variables: Sway velocity–Self‐reported kama muta	*N*	Spearman's rho	*p*	Fisher's *z*	*SE* effect size	95% CI [LL, UP]
Video 0 (neutral/calm)	79	−0.035	0.758	−0.035	0.114	[–0.25, 0.19]
Video 1 (kama muta)	72	−0.159	0.183	−0.160	0.121	[–0.38, 0.08]
Video 2 (kama muta)	77	0.178	0.121	0.180	0.117	[–0.05, 0.39]
Video 3 (kama muta)	78	0.117	0.308	0.117	0.116	[–0.11, 0.33]
Video 4 (kama muta)	78	0.217	0.056	0.221	0.117	[–0.01, 0.42]
Video 5 (kama muta)	78	0.109	0.341	0.110	0.116	[–0.12, 0.32]

*Note*: Table 2 reports the results of Spearman's rank‐order correlations between sway velocity and self‐reported experiences of being moved for six video stimuli: one neutral (Video 0) and five kama muta videos (Videos 1–5). Spearman's rho, *p*‐values, and effect sizes are reported. *N* refers to the number of participants in each condition. Effect size is expressed using Fisher's *z*, with the corresponding standard error (*SE*).

When results for mean amplitude and sway velocity were compared, the correlations were broadly similar: neither of the sway metric parameters consistently demonstrated significant correlations with self‐reported experiences of being moved.

## 4 Discussion

The present study aimed to investigate whether the emotional experience of being moved corresponded with measurable changes in postural sway. The results partially supported H1a. While there was no significant difference in mean amplitude between the kama muta and neutral/calm conditions, a significant reduction in sway velocity was observed during the kama muta condition compared to the neutral/calm condition. For H1b, contrary to our hypothesis that sway metrics would increase in the second half of the recording due to intensifying emotional arousal, both mean amplitude and sway velocity decreased significantly. This unexpected decrease suggests that kama muta may promote a stabilizing effect over time, potentially reflecting physical grounding as the emotional experience deepens. In the neutral condition, sway velocity showed no significant change, reinforcing the specificity of this stabilizing effect to kama muta. H2 was not supported, as no significant correlations were found between self‐reported moving experiences and either sway velocity or mean amplitude.

The difference between sway amplitude and velocity provides insight into how kama muta affected postural control in our study. Sway amplitude refers to how far the CoP shifts over time, reflecting the magnitude of postural sway. A larger amplitude can reflect greater instability or exploratory balance adjustments, whereas a smaller amplitude suggests more constrained movements. In contrast, sway velocity reflects the speed of postural adjustments, providing information about the dynamism and responsiveness of postural control mechanisms (Duarte and Freitas 2010). Faster sway velocity may indicate instability, as the body makes frequent corrections, while slower velocity suggests smoother, more controlled balance. A reduction in sway velocity without a significant change in mean amplitude suggests that while participants did not sway less in distance, their movement became slower and more controlled during the emotional experience of kama muta. This finding aligns with previous research indicating that emotional states can influence motor control and postural stability. For instance, studies have shown that certain emotions can lead to a “freezing” response, characterized by reduced sway velocity and amplitude, primarily in response to negative emotions such as fear or threat (Facchinetti et al. 2006; Roelofs et al. 2010). However, our findings question whether this reduction should universally be interpreted as freezing, as we observed reduced sway velocity during a positive emotional state without a concurrent reduction in sway amplitude.

Our results extend the understanding of postural control during positive, self‐transcendent emotions like kama muta. The reduction in sway velocity may reflect a stabilizing effect on postural control, indicating that participants become more physically grounded during the emotional experience. This stabilizing effect could be a manifestation of the body's response to intense social and emotional bonding, as kama muta is associated with feelings of unity and connection (Fiske 2020).

The lack of a significant difference in mean amplitude between the kama muta and neutral conditions suggests that while the speed of postural adjustments decreased, the overall extent of sway did not change. This indicates that kama muta specifically influences the dynamics of postural control (i.e., how quickly adjustments are made: sway velocity) rather than the magnitude of postural sway (i.e., how far the body moves from the center: mean amplitude).

Contrary to our initial expectation that sway velocity and mean amplitude would increase in the second half of the emotional stimuli due to intensifying emotional arousal, we observed a significant decrease in both parameters during the second half of the kama muta condition. In the neutral condition, only mean amplitude decreased significantly, with no significant change in sway velocity. The decrease in sway velocity and mean amplitude over time during the kama muta condition reinforces the notion of a stabilizing effect on postural control. As the emotional content of the videos intensified, participants exhibited reduced postural sway; their sway patterns became slower and more confined, reflecting an overall stillness. This pattern suggests that kama muta could lead to a physical grounding or stillness as the emotional experience deepens.

This aligns with previous findings by Stins et al. (2011), who reported that emotional stimuli requiring internal focus and reflection decreased postural sway, leading to greater stability. Fredrickson and Levenson (1998) also demonstrated that positive emotions can speed recovery from the cardiovascular sequelae of negative emotions, suggesting a regulatory effect on physiological responses. Thus, our results can be interpreted as evidence that kama muta promotes adaptive benefits, enhances social cohesion, and fosters well‐being through emotional and physical mechanisms. The reduction in mean amplitude during the neutral condition's second half may be attributed to participants becoming more accustomed to the experimental setup or experiencing general fatigue, leading to decreased movement. However, the lack of significant change in sway velocity during the neutral condition suggests that the stabilizing effect observed in the kama muta condition is specifically related to the emotional content of the stimuli.

The absence of significant correlations between self‐reported experiences of being moved and postural sway metrics suggests that individual subjective reports did not directly correspond to changes in sway velocity or mean amplitude. This finding may be due to several factors. First, using a single‐item self‐report measure may not have captured the full depth and nuances of the kama muta experience; this is a risk we anticipated and took into account anyway, as we decided to pilot this research. Multidimensional scales assessing various facets of emotion might provide a more sensitive measure (Zickfeld et al. 2019). Second, individual differences in emotional expressiveness and embodiment could attenuate the relationship between subjective experience and physiological responses. Some individuals may experience strong emotions internally without recognizing alterations in motor control, while others may show pronounced physical reactions (Mauss et al. 2005). This individual variability in how participants interpret “being moved or touched” could potentially disturb any direct correlation. Third, the floor effects in the CoP measures and potential variability in participants’ baseline postural control may have limited the ability to detect subtle correlations with self‐reported emotions. Fourth, the methodological framework and structure of measurement employed in this exploratory study may have shaped the pattern of results, influencing not only what was detectable but also how the emotional dynamics of kama muta were captured. Given our central research question: whether the body moves when we feel moved, it remains an open consideration whether the methodological choices made here were sensitive enough to detect the full spectrum of embodied emotional responses. Also, sway velocity and amplitude are only two measurements of postural sway. Structural parameters like sample entropy in the anterior–posterior or medio–lateral direction could have been analyzed. Those structural parameters assess the regularity of the CoP time series.

### 4.1 Limitations of the Study and Future Directions

The study of the relationship between body movement and kama muta is just at the beginning. Even if it seems promising, several limitations should be acknowledged. As a first exploratory step, a single item was used to investigate kama muta. Subsequent investigations should consider using validated multi‐item scales, such as the Kama Muta Multiplex Scale (Zickfeld et al. 2019), to capture the complexity of the emotional experience. Furthermore, the sample consisted primarily of university students, which may limit the generalizability of the findings to broader populations. Future research could include more diverse samples to enhance the representativeness of the results. The standing posture required for the force plate measurements may have constrained natural movements, potentially influencing the expression of embodied emotional responses. For example, participants were instructed to be still and maintain their posture during the trials. Future studies could explore alternative setups.

While the emotional videos used in this study were validated to evoke kama muta, the variability in individual responses to these stimuli could have influenced the results. Emotional intensity and embodiment may differ depending on personal relevance or cultural context. Employing a wider range of stimuli or personalized content could enhance the robustness of the findings. Examining the role of individual differences in emotional embodiment, such as interoceptive awareness or trait emotional expressiveness, could also help explain variability in responses.

Although sway velocity and amplitude are valuable indicators of postural control, they provide a limited perspective on motor responses. Other metrics, including physiological measures such as skin conductance, movement synchronization, or muscle activation patterns, measured by electromyography, could offer a more comprehensive understanding of the embodied aspects of kama muta. Additionally, incorporating continuous, moment‐to‐moment measures of emotional experience and postural sway could help capture and understand postural sway changes during the kama muta experience.

Finally, examining the stimuli in sequential segments, via time‐series analyses, could uncover distinctions in how kama muta unfolds over time. Such granularity was beyond the scope of the current study. Future research may further integrate these advanced analytical approaches to investigate this embodied experience's temporal dynamics.

## 5 Conclusion

This study provides evidence that the emotional experience of being moved is associated with measurable changes in postural sway, specifically a reduction in sway velocity. The findings suggest that kama muta stabilizes postural control, increasing physical grounding during the emotional experience. The decrease in sway velocity and mean amplitude over time indicates that this stabilizing effect intensifies as the emotional experience deepens.

While no direct correlation was found between self‐reported emotional intensity and sway metrics, the study highlights the complex interplay between emotions and motor responses. These findings contribute to understanding emotional embodiment and suggest that kama muta promotes postural stability, reflecting adaptive mechanisms that facilitate social bonding and emotional processing. Furthermore, analogous to how physical touch and contact stabilize postural sway, the experience of kama muta may similarly contribute to postural stability. However, this interpretation remains speculative and warrants further investigation in future research.

Given kama muta's role in fostering social bonding, future research could explore its therapeutic applications, particularly in interventions to enhance interpersonal connection or address social isolation.

## Author Contributions

**Maria Meisel**: conceptualization, investigation, data curation, writing – original draft, formal analysis, methodology. **Philipp Hofmann**: methodology, writing – review and editing, formal analysis. **Petra Jansen**: conceptualization, funding acquisition, methodology, writing – review and editing, supervision.

## Disclosure

This study was preregistered at OSF (registration DOI https://doi.org/10.17605/OSF.IO/P8ZQF).

## Ethics Statement

This study was approved by the Ethical Committee of the University of Regensburg (Approval Number: 22–2892_1‐101).

## Consent

Written informed consent was obtained from all participants.

## Conflicts of Interest

The authors declare no conflicts of interest.

## Peer Review

The peer review history for this article is available at https://publons.com/publon/10.1002/brb3.70897.

## Data Availability

Data supporting this study is available on the Open Science Framework: https://doi.org/10.17605/OSF.IO/P8ZQF.

## References

[brb370897-bib-0001] Appelbaum, M. , H. Cooper , R. B. Kline , E. Mayo‐Wilson , A. M. Nezu , and S. M. Rao . 2018. “Journal Article Reporting Standards for Quantitative Research in Psychology: The APA Publications and Communications Board Task Force Report.” American Psychologist 73, no. 1: 3–25. 10.1037/amp0000191. 29345484

[brb370897-bib-0002] Barrett, L. F. 2018. How Emotions Are Made: The Secret Life of the Brain. Paperback edition. PAN Books.

[brb370897-bib-0003] Braine, A. , and F. Georges . 2023. “Emotion in Action: When Emotions Meet Motor Circuits.” Neuroscience & Biobehavioral Reviews 155: 105475. 10.1016/j.neubiorev.2023.105475. 37996047

[brb370897-bib-0004] De Gelder, B. , A. W. De Borst , and R. Watson . 2015. “The Perception of Emotion in Body Expressions.” WIREs Cognitive Science 6, no. 2: 149–158. 10.1002/wcs.1335. 26263069

[brb370897-bib-0005] Doyle, R. J. , E. T. Hsiao‐Wecksler , B. G. Ragan , and K. S. Rosengren . 2007. “Generalizability of Center of Pressure Measures of Quiet Standing.” Gait & Posture 25, no. 2: 166–171. 10.1016/j.gaitpost.2006.03.004. 16624560

[brb370897-bib-0006] Duarte, M. , and S. M. S. F. Freitas . 2010. “Revision of Posturography Based on Force Plate for Balance Evaluation.” Revista Brasileira De Fisioterapia 14, no. 3: 183–192. 10.1590/S1413-35552010000300003. 20730361

[brb370897-bib-0007] Duarte, M. , and R. N. Watanabe . 2023. “Postural Control in Humans: Theories, Modeling, and Quantification.” In Current Trends in Biomedical Engineering, edited by C. B. Lombello and P. A. Da Ana , 17–34. Springer International Publishing. 10.1007/978-3-031-38743-2_2.

[brb370897-bib-0008] Facchinetti, L. D. , L. A. Imbiriba , T. M. Azevedo , C. D. Vargas , and E. Volchan . 2006. “Postural Modulation Induced by Pictures Depicting Prosocial or Dangerous Contexts.” Neuroscience Letters 410, no. 1: 52–56. 10.1016/j.neulet.2006.09.063. 17056179

[brb370897-bib-0009] Faul, F. , E. Erdfelder , A.‐G. Lang , and A. Buchner . 2007. “G*Power 3: A Flexible Statistical Power Analysis Program for the Social, Behavioral, and Biomedical Sciences.” Behavior Research Methods 39, no. 2: 175–191. 10.3758/BF03193146. 17695343

[brb370897-bib-0010] Fiske, A. P. 2020. Kama Muta: Discovering the Connecting Emotion. Routledge.

[brb370897-bib-0011] Fredrickson, B. L. , and R. W. Levenson . 1998. “Positive Emotions Speed Recovery From the Cardiovascular Sequelae of Negative Emotions.” Cognition and Emotion 12, no. 2: 191–220. 10.1080/026999398379718. 21852890 PMC3156608

[brb370897-bib-0012] Gea, J. , M. A. Muñoz , I. Costa , L. F. Ciria , J. G. V. Miranda , and P. Montoya . 2014. “Viewing Pain and Happy Faces Elicited Similar Changes in Postural Body Sway.” PLoS ONE 9, no. 8: e104381. 10.1371/journal.pone.0104381. 25093727 PMC4122445

[brb370897-bib-0013] Hall, K. J. , K. Van Ooteghem , and W. E. McIlroy . 2023. “Emotional State as a Modulator of Autonomic and Somatic Nervous System Activity in Postural Control: A Review.” Frontiers in Neurology 14: 1188799. 10.3389/fneur.2023.1188799. 37719760 PMC10500443

[brb370897-bib-0014] Hillman, C. H. , K. S. Rosengren , and D. P. Smith . 2004. “Emotion and Motivated Behavior: Postural Adjustments to Affective Picture Viewing.” Biological Psychology 66, no. 1: 51–62. 10.1016/j.biopsycho.2003.07.005. 15019170

[brb370897-bib-0015] Hofmann, P. , L. Jost , and P. Jansen . 2021. “Embodied Mental Rotation—Does It Affect Postural Stability?” Journal of Motor Behavior 55, no. 2: 202–219. 10.1080/00222895.2022.2151970. 36473703

[brb370897-bib-0016] Horslen, B. C. , and M. G. Carpenter . 2011. “Arousal, Valence and Their Relative Effects on Postural Control.” Experimental Brain Research 215, no. 1: 27–34. 10.1007/s00221-011-2867-9. 21947171

[brb370897-bib-0017] İyilikci, E. A. , M. Boğa , E. Yüvrük , Y. Özkılıç , O. İyilikci , and S. Amado . 2023. “An Extended Emotion‐Eliciting Film Clips Set (EGEFILM): Assessment of Emotion Ratings for 104 Film Clips in a Turkish Sample.” Behavior Research Methods 56, no. 2: 529–562. 10.3758/s13428-022-02055-4. 36737582

[brb370897-bib-0018] JASP Team . 2024. “JASP (Version 0.18.3).” https://jasp‐stats.org/.

[brb370897-bib-0020] Lang, P. J. 1995. “The Emotion Probe: Studies of Motivation and Attention.” American Psychologist 50, no. 5: 372–385. 10.1037/0003-066X.50.5.372. 7762889

[brb370897-bib-0021] Lelard, T. , P. Krystkowiak , B. Montalan , et al. 2014. “Influence of Postural Threat on Postural Responses to Aversive Visual Stimuli.” Behavioural Brain Research 266: 137–145. 10.1016/j.bbr.2014.02.051. 24631393

[brb370897-bib-0022] Maki, B. E. , and W. E. McIlroy . 1996. “Influence of Arousal and Attention on the Control of Postural Sway.” Journal of Vestibular Research 6, no. 1: 53–59. 10.3233/VES-1996-6107. 8719510

[brb370897-bib-0023] Mauss, I. B. , R. W. Levenson , L. McCarter , F. H. Wilhelm , and J. J. Gross . 2005. “The Tie That Binds? Coherence Among Emotion Experience, Behavior, and Physiology.” Emotion 5, no. 2: 175–190. 10.1037/1528-3542.5.2.175. 15982083

[brb370897-bib-0024] Meier, B. P. , S. Schnall , N. Schwarz , and J. A. Bargh . 2012. “Embodiment in Social Psychology.” Topics in Cognitive Science 4, no. 4: 705–716. 10.1111/j.1756-8765.2012.01212.x. 22777820

[brb370897-bib-0025] Peirce, J. , J. R. Gray , S. Simpson , et al. 2019. “PsychoPy2: Experiments in Behavior Made Easy.” Behavior Research Methods 51, no. 1: 195–203. 10.3758/s13428-018-01193-y. 30734206 PMC6420413

[brb370897-bib-0026] Prieto, T. E. , J. B. Myklebust , R. G. Hoffmann , E. G. Lovett , and B. M. Myklebust . 1996. “Measures of Postural Steadiness: Differences Between Healthy Young and Elderly Adults.” IEEE Transactions on Biomedical Engineering 43, no. 9: 956–966. 10.1109/10.532130. 9214811

[brb370897-bib-0027] Python Software Foundation . 2021. “Python (Version 3.9.6).” https://www.python.org.

[brb370897-bib-0028] Quijoux, F. , A. Nicolaï , I. Chairi , et al. 2021. “A Review of Center of Pressure (COP) Variables to Quantify Standing Balance in Elderly People: Algorithms and Open‐Access Code.” Physiological Reports 9, no. 22: e15067. 10.14814/phy2.15067. 34826208 PMC8623280

[brb370897-bib-0029] R Core Team . 2023. R: A Language and Environment for Statistical Computing. R Foundation for Statistical Computing. https://www.R‐project.org/.

[brb370897-bib-0030] Roelofs, K. , M. A. Hagenaars , and J. Stins . 2010. “Facing Freeze: Social Threat Induces Bodily Freeze in Humans.” Psychological Science 21, no. 11: 1575–1581. 10.1177/0956797610384746. 20876881

[brb370897-bib-0031] Rottenberg, J. , R. D. Ray , and J. J. Gross . 2007. “Emotion Elicitation Using Films.” In Handbook of Emotion Elicitation and Assessment, edited by J. A. Coan and J. J. B. Allen , 9–28. Oxford University Press. 10.1093/oso/9780195169157.003.0002.

[brb370897-bib-0032] Sapir, E. 1956. Culture, Language and Personality: Selected Essays. Edited by D. G. Mandelbaum . University of California Press.

[brb370897-bib-0033] Schönfeld, L.‐M. , and L. Wojtecki . 2019. “Beyond Emotions: Oscillations of the Amygdala and Their Implications for Electrical Neuromodulation.” Frontiers in Neuroscience 13: 366. 10.3389/fnins.2019.00366. 31057358 PMC6482269

[brb370897-bib-0034] Schubert, T. W. , J. H. Zickfeld , B. Seibt , and A. P. Fiske . 2018. “Moment‐to‐Moment Changes in Feeling Moved Match Changes in Closeness, Tears, Goosebumps, and Warmth: Time Series Analyses.” Cognition and Emotion 32, no. 1: 174–184. 10.1080/02699931.2016.1268998. 28024440

[brb370897-bib-0035] Shopovski, J. 2024. “Generative Artificial Intelligence, AI for Scientific Writing: A Literature Review.” Preprint, Preprints.org, June 3. 10.20944/preprints202406.0011.v1.

[brb370897-bib-0036] Stins, J. F. , and P. J. Beek . 2007. “Effects of Affective Picture Viewing on Postural Control.” BMC Neuroscience 8, no. 1: 83. 10.1186/1471-2202-8-83. 17916245 PMC2082031

[brb370897-bib-0037] Stins, J. F. , M. Roerdink , and P. J. Beek . 2011. “To Freeze or Not to Freeze? Affective and Cognitive Perturbations Have Markedly Different Effects on Postural Control.” Human Movement Science 30, no. 2: 190–202. 10.1016/j.humov.2010.05.013. 20727608

[brb370897-bib-0038] Vaezmousavi, M. , and M. Osanlu . 2011. “Skin Conductance Level Predicts Performance in a Balance Task.” World Journal of Sports Science 4, no. 2: 139–143.

[brb370897-bib-0039] Vicon Motion Systems . n.d. “Vicon Nexus (Version 2.13.0).” https://www.vicon.com.

[brb370897-bib-0040] Whorf, B. L. 1956. Language, Thought, and Reality: Selected Writings of Benjamin Lee Whorf, edited by J. B. Carroll . MIT Press.

[brb370897-bib-0042] Winter, D. 1995. “Human Balance and Posture Control During Standing and Walking.” Gait & Posture 3, no. 4: 193–214. 10.1016/0966-6362(96)82849-9.

[brb370897-bib-0043] Zickfeld, J. H. , P. Arriaga , S. V. Santos , T. W. Schubert , and B. Seibt . 2020. “Tears of Joy, Aesthetic Chills and Heartwarming Feelings: Physiological Correlates of Kama Muta.” Psychophysiology 57, no. 12: e13662. 10.1111/psyp.13662. 32786039

[brb370897-bib-0044] Zickfeld, J. H. , T. W. Schubert , B. Seibt , et al. 2019. “Kama Muta: Conceptualizing and Measuring the Experience Often Labelled Being Moved Across 19 Nations and 15 Languages.” Emotion 19, no. 3: 402–424. 10.1037/emo0000450. 29888936

